# Iodoform as a Dressing for Wounds

**Published:** 1882-02

**Authors:** 


### Iodoform as a Dressing for Wounds.

Mikulicz (in *Wiener Med. Wochenschrift*, 1881, No. 23) gives results of the use of iodoform in Billroth's wards. He claims that it is in antiseptic qualities equal to carbolic acid, is more easily used, and less apt to cause constitutional disturbance by absorption. Symptoms of poisoning are, however, seen in rare cases, and in the *Deutsche Med. Woch.*, 1881, No. 34, A. Henry describes two fatal cases. The symptoms are of the narcotico-irritant type.

In open wounds the iodoform is sprinkled on the surface and covered with lint and gutta-percha tissue, fixed by a bandage. The results have been very satisfactory; the dressings require changing but seldom, discharge is slight, decomposition never occurs, and there is rapid formation of healthy granulations. In incised wounds healing is even more certain than with carbolic acid, and there is much less fear of absorption causing constitutional disturbance.

Wounds implicating mucous surfaces, as of the mouth or rectum, are usually very difficult to treat antisceptically. In such cases iodoform, applied on gauze compresses, has been found to completely prevent offensive smell, and to cause no discomfort to the patients.

In a case of removal of an abdominal tumor iodoform was sprinkled into the cavity, and the wound closed at once. The patient recovered without a bad symptom.

In septic, gangrenous, or sloughing wounds the results were especially satisfactory. Sprinkling with iodoform removed all smell in from four to six hours, and the wounds healed rapidly and without discharge, even in some cases where severe constitutional symptoms had already appeared.

In strumous diseases iodoform is said to give such brilliant results as almost to entitle it to the rank of a specific. (See also V. Mosetig-Moorhof in *Wien. Med. Woch.*, 1881, No. 13.) Fungating ulcers with spreading undermined edges and offensive discharge healed rapidly and completely under a thick layer of iodoform.

In lupus also its effects are gratifying. Riehl (*Wien. Med. Woch.*, 1881, No. 19) gives the results of twenty cases in Kaposi's clinique. The epidermis, when necessary, having been removed by the application of five to ten per cent. solution of caustic potash, the iodoform is laid on in a layer several millimetres thick, and fixed as above described. On removal of the dressings in from three to eight days the disease is found completely removed, redness and swelling gone, and the sore skinned over.

In deep wounds, when the powder would be difficult to apply, Mikulicz recommends pencils composed of one part of iodoform to two of cacao butter, and for injection a twenty per cent. ethereal solution. The smell of the drug can be overcome by adding one minim bergamot to ten grains of the iodoform, or moistening with an ethereal or alcoholic extract of Tonquin bean. Local irritation can be effectually prevented by previously oiling the sound skin near where the iodoform is to be applied. —*Centralbl. f. Chir.*, 1881, Nos. 32 and 39.

